# Chinese Survey on Enhanced Recovery after Surgery and Thromboprophylaxis Following Arthroplasty

**DOI:** 10.1111/os.12705

**Published:** 2020-06-03

**Authors:** Xi‐sheng Weng, Juan Liu, Duo Wu

**Affiliations:** ^1^ Department of Orthopedics Peking Union Medical College Hospital Beijing China; ^2^ Medical Affairs Department Pfizer Inc Shanghai China; ^3^ Medical Affairs Department Pfizer Inc Guangzhou China

**Keywords:** Arthroplasty, Enhanced recovery after surgery, Thromboprophylaxis, Venous thromboembolism

## Abstract

**Objective:**

To examine the current perspectives of enhanced recovery after surgery (ERAS) and the clinical practice applications of important ERAS principles among Chinese orthopaedic surgeons.

**Methods:**

This was a cross‐sectional study using an online survey that was completed between November and December 2018. A 16‐item online questionnaire regarding the experiences of ERAS, perceptions of methods, and durations and concerns of venous thromboembolism (VTE) prophylaxis was sent to 2000 orthopaedic surgeons nationwide, and 1720 (86%) surgeons responded. Statistical analyses were conducted to assess all respondents' results and to compare differences among subgroups that were stratified according to city and hospital level, as well as their professional title.

**Results:**

According to the results of the survey, ERAS awareness was high (65.1%) and most surgeons recognized the importance of thromboprophylaxis. However, the timing of ERAS was not consistent, with 22.8%, 31.9%, and 37.7% of surgeons choosing to initiate pharmaceutical prophylaxis within <6 h, 6–12 h, and 12–24 h after surgery, respectively. Low‐molecular‐weight heparin was mainly selected during hospitalization, and new oral anticoagulants (NOACs) were the first choice after discharge. Regarding postoperative antithrombotic therapy, particularly when combined with analgesics, the potential bleeding risk was mostly considered (80.0%)Tranexamic acid was believed to have no effect on the timing of NOAC therapy initiation (56.2%). Most of the above outcomes were influenced by the hospital level and professional title of the surgeon. Surgeons who had higher awareness on ERAS and better adhered to the guidelines were from higher‐level hospitals as well as had more advanced professional titles. City level partly might influence their practice but not impact surgeons' awareness.

**Conclusions:**

The awareness and perception of the concept of ERAS and prophylactic antithrombotic regimens remain different among Chinese orthopaedic surgeons in different level cities and with various professional titles. Continuing medical educations (CME) on VTE prophylaxis is needed for improving the quality of health care in China.

## Introduction

The concept of enhanced recovery after surgery (ERAS) was first proposed by Kehlet in the 1990s^1^. The goals of ERAS are to promote faster recovery, reduce postoperative complications, shorten the length of stay (LOS), reduce economic burden, and improve patient satisfaction using evidence‐based methods^2^. When it was first applied in gastrointestinal surgery, successful implementation of ERAS protocols and the adherence to ERAS protocols that achieved satisfactory outcomes. Since then, ERAS has been adopted in various fields such as general surgery, cardiothoracic surgery, orthopaedics, gynecology, and urology^3^, ^4^, ^5^, ^6^.

In recent years, ERAS has demonstrated remarkable results in total hip arthroplasty/knee arthroplasty (THA/TKA)^7^. A meta‐analysis showed that ERAS could reduce the LOS and incidence of complications in patients undergoing THA/TKA^8^. For surgeons, complications including the risk of venous thromboembolism (VTE) or bleeding are the most important issues in the postoperative period. These complications might increase patients' burden, lower patient satisfaction, and delay discharge. This problems could further strain the relationship between patients and surgeons, particularly in the current healthcare environment in China. Besides improvements in orthopaedic surgical techniques, drug administration before and after surgery is critical for balancing the risks of VTE and bleeding.

Although the understanding of the concept of ERAS is gradually increasing in China, it has only been implemented in a few tertiary hospitals. Surgeons in provincial or municipal hospitals might have limited understanding and experience of ERAS management, and these surgeons might be unsure about the current state of ERAS implementation. For these surgeons, the administration of drugs for thromboprophylaxis might be nonstandard. Previous studies reported numerous inconsistencies in the understanding of ERAS and drug administration in the field of bariatric surgery^9^. Additionally, no previous survey has assessed the understanding of ERAS and thromboprophylaxis following joint replacement among surgeons in China. Such study findings would be helpful to support ERAS implementation and improve healthcare quality following major orthopaedic surgery.

The present study aimed to investigate the understanding of ERAS and VTE prevention among orthopaedic surgeons from different backgrounds and related factors such as the method, duration, and consideration of pharmacologic prophylaxis.

## Materials and Methods

### *Study Design*

A total of 2000 orthopaedic surgeons selected by stratified random sampling from 31 provincial administrative regions in mainland China were enrolled in the current study.

### *Questionnaire Design*

A 16‐item questionnaire was employed to gather the awareness, perceptive, and practice of surgeons after THA/TKA surgery. The questionnaire also collected information about the different city and hospital levels, as well as the surgeon's professional title, that was used in stratification for further subgroup analysis. The survey was anonymous, and a unified code was used for data input and statistics. The questionnaire was designed following five dimensions: knowledge of ERAS and the management of antithrombotic therapy, timing and duration of anticoagulation, choice and concern of antithrombotic drugs in practice, combined medication, and the need of antithrombotic prophylactic management (Table 1). A quick‐response code was scanned by using a mobile terminal to enter each questionnaire, and the system required responses to all questions for result submission.

**TABLE 1:** Questionnaire and the results from surgeons

Dimension	No.	Question	Options		Count	Total number
Cognition of ERAS and antithrombotic management	1	For ERAS, please choose the most appropriate description	I have heard of ERAS and have certain understanding about it		666	*n* = 1720
			My Department are planning to run an ERAS program in near future		453	
			The ERAS project is under way in my department		504	
			I have not heard of ERAS and have no idea what it is		97	
	2	How do you think about the implementation of ERAS project?	Improves efficiency by standardized perioperative management; support to continue ERAS program		1550	*n* = 1623
			Increased workload, there is no need to continue to carry out		73	
	3	What do you think of the effectiveness of ERAS project?	Postoperative rehabilitation was significantly improved		1207	*n* = 1623
			Somehow helpful to patients' recovery, but no significant changes to postoperative complication or length of stay in hospital		399	
			No improvement in postoperative rehabilitation was observed		17	
	4	In your opinion, the importance of optimizing perioperative thrombosis management for patients' postoperative rehabilitation	The effect of optimizing perioperative thrombosis management on postoperative rehabilitation is limited		487	*n* = 1720
			Optimizing perioperative thromboembolism management has a certain effect on postoperative rehabilitation, but it is not a key factor		464	
			Optimizing perioperative thrombosis management is very important and a prerequisite for postoperative rehabilitation		769	
	5	Patients after arthroplasty are at high risk of venous thrombosis, and all patients need to take necessary preventive measures.	Yes		1644	*n* = 1720
			No		76	
Timing and duration of anticoagulation	6	Usually, the timing of anticoagulant use after surgery	Within 6 h		342	*n* = 1497
			6‐12 h		478	
			12‐24 h		564	
			Exceeding 24h		113	
	7	For patients undergoing total knee arthroplasty (TKA), how long do you use drugs to prevent VTE?	Within 7 days		369	*n* = 1720
			7—10 days		387	
			10—14 days		964	
	8	For patients undergoing total hip arthroplasty (THA), how long do you use drugs to prevent VTE?	Within 1 week		291	*n* = 1720
			1–3 weeks		466	
			3–4 weeks		438	
			4–5 weeks		525	
	9	What are the reasons for the failure to anticoagulants for the recommended length of time in accordance with the guidelines?	The patient condition is different and there is no need to follow the course of treatment		401	*n* = 1720
			Usually no clinical symptoms of VTE were found		224	
			Postoperative VTE complications are low‐probability clinical events		340	
			Possible side effects of anticoagulation therapy after discharge cannot be monitored in time		755	
Choice and concern of antithrombotic drugs in practice	10	What drugs do you usually use to prevent VTE during hospitalization? (multiple choices)	LMWHs	Yes	1391	*n* = 1720
				No	329	
			NOAC	Yes	810	
				No	910	
			Vitamin K antagonist	Yes	274	
				No	1446	
			Antiplatelet drugs	Yes	356	
				No	1364	
	11	What drugs do you usually use to prevent VTE after discharge? (multiple choices)	LMWHs	Yes	622	*n* = 1720
				No	1098	
			NOAC	Yes	894	
				No	826	
			Vitamin K antagonist	Yes	350	
				No	1370	
			Antiplatelet drugs	Yes	572	
				No	1148	
	12	Do you have the following concerns about injectable anticoagulants in post‐operative antithrombotic management? (multiple choices)	Poor patient compliance	Yes	909	*n* = 1586
				No	677	
			Thrombocytopenia (HIT) risk	Yes	726	
				No	860	
			The risk of bleeding is relatively high	Yes	1031	
				No	555	
			VTE preventive effect is not good	Yes	530	
				No	1056	
			Therapeutic effect is unpredictable	Yes	420	
				No	1166	
			None of the above descriptions are accepted	Yes	134	
				No	1586	
Combined medication	13	Effect of the use of tranexamic acid on the initial administration time of NOAC	No impact		966	*n* = 1720
			In advance		489	
			Delayed		265	
	14	Whether the effect of analgesics on the risk of bleeding will be considered when patients use drugs to prevent thrombosis after operation	Yes		1374	*n* = 1720
			No		346	
Demand for antithrombotic management in ERAS	15	Is it necessary to conduct research to evaluate the effect of antithrombotic drugs on postoperative wound complications?	Strongly agree		918	*n* = 1720
			Agree		626	
			Slightly disagree		97	
			Disagree		36	
			Uncertain		43	
	16	Is it necessary to conduct a follow‐up study to evaluate the quality of preventive thrombosis treatment after TKA/THA discharged from hospital?	Strongly agree		1022	*n* = 1720
			Agree		594	
			Slightly disagree		76	
			Disagree		9	
			Uncertain		19	

### *Data Analysis and Subgroup Definition*

Because the questionnaire comprised choice‐ or judgment‐based questions, all study data are presented as frequencies. Percentage (%) values and chi‐square tests were used to show the proportions of the options chosen among the subgroups.

The subgroup definition was conducted according to the city level (first‐tier, second‐tier, or other‐tier city refer to the population; for example, first‐tier cities include Shanghai, Beijing, and Guangzhou, and second‐tier cities include some provincial capitals like Hangzhou, Nanjing), hospital level (tertiary, secondary, or community hospital), or surgeons' professional title (chief physician, deputy chief physician, attending physician, resident physician, or graduate student) .

Statistical analysis was performed using SAS 9.5 software (SAS Institute, Cary, North Carolina, USA). A *P*‐value <0.05 was considered significant, and Bonferroni correction was used for multiple comparisons among the subgroups.

## Results

### *General Information*

**FIG 1:**
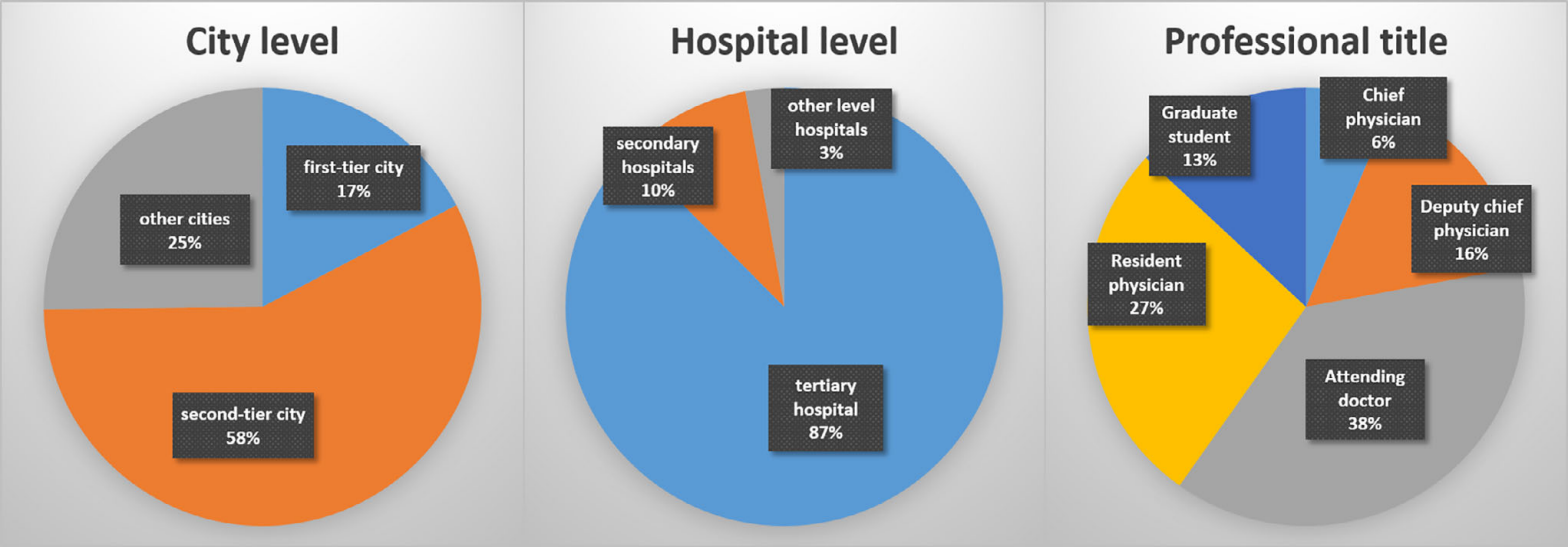
Basic information of surgeons.

A total of 1720 (86.0%) valid questionnaires were obtained and analyzed, among which 298 (17.3%) were from surgeons in first‐tier cities, 989 (57.5%) in second‐tier cities, and 433 (25.2%) in other‐tier cities (Fig. 1). Most surgeons were from tertiary hospitals (1506/1720, 87.6%), which are the most advanced hospitals in China. Regarding professional title, 110 (6.4%) surgeons were chief physicians, 270 (15.7%) were deputy chief physicians, 649 (37.7%) were attending physicians, 466 (27.1%) were resident physicians, and 225 (13.1%) were graduate students.

### *Perceptive of* *ERAS* *and Administration of Antithrombotic Medications*

Most surgeons (1119/1720, 65.1%; Table 1) were aware of the concept of ERAS or had planned to implement ERAS programs in their departments and believed that ERAS improves efficiency and was helpful in practice. The timing of ERAS was inconsistent, with 22.8% (342/1497), 31.9% (478/1497), and 37.7% (564/1497) of surgeons choosing to initiate anticoagulant therapy at <6 h, 6–12 h, and 12–24 h after surgery, respectively. However, over half of surgeons believed that VTE prevention for 10–14 days was necessary. About 31% surgeons would prescribe 4–5 weeks VTE prevention after THA.

### *Choice of Antithrombotic Drugs in Practice and Concerns*

Most (80.9%) surgeons prescribed low‐molecular‐weight heparin (LMWH), whereas half chose new oral anticoagulants (NOACs).

However, regarding use of medicines following discharge, the proportion of surgeons who chose LMWH noticeably decreased to <36.2%, whereas the proportions of surgeons who chose NOACs, vitamin K antagonists, and antiplatelet drugs slightly increased. Bleeding risk (65.0%, 1031/1586) was the greatest concern when choosing postoperative antithrombotic therapies, followed by patient adherence (57.3%, 909/1586) and thrombocytopenia risk (45.8%, 726/1586).

Furthermore, over half of the surgeons (966/1720, 56.2%) believed that tranexamic acid (TXA) did not affect the initial NOAC administration time. Moreover, 79.9% (1374/1720) considered the effect of analgesics on bleeding risk.

### *Need to Improve Antithrombotic Management of Patients*

For the management of antithrombotic therapy, most surgeons thought that it was essential to perform studies evaluating the effects of antithrombotic drugs on wound complications and assessing the quality of preventive thrombosis treatment after discharge.

### *Subgroup Analysis and Comparisons*

Among the five question dimensions, city level mainly influenced therapeutic use of anticoagulants, including the timing of initiation and duration, as well as the different types of medications chosen (Table 2). However, hospital level and professional title also substantially influenced all these dimensions, with at least one statistically significant difference in each dimension. Higher percentages have emphasized the significance of ERAS and thromboprophylaxis strategies among the surgeons are in tertiary hospitals or have the title of chief physicians.

**TABLE 2:** Subgroups analysis of answer proportion by different stratification

Question no.	Different city level	Different hospital level	Professional title
1	0.348	<0.001	<0.001
2	0.441	0.608	0.735
3	0.639	0.244	0.368
4	0.278	0.020	0.020
5	0.143	0.130	0.103
6	0.013	0.043	<0.001
7	0.019	<0.001	<0.001
8	0.114	0.002	<0.001
9	0.225	0.047	0.136
10	0.001	<0.001	<0.001
11	0.022	0.007	<0.001
12	0.337	0.148	0.004
13	0.362	0.001	<0.001
14	0.060	0.073	0.596
15	0.464	0.065	0.055
16	0.349	<0.001	0.037

Chi‐square test were used to analyze the difference of the options were chosen among the subgroups, divided according to the stratification. A *P*‐value of <0.05 was considered significant, and Bonferroni correction was used for multiple comparisons among the subgroups.

The responses to two questions concerning implementation of an ERAS protocol and effectiveness were consistent in all subgroup comparisons (Table 2). Additionally, understanding about the effects of analgesics and the opinion on the necessity to conduct further studies to evaluate the effects of antithrombotic drugs on wound complications were consistent. The responses to other questions differed in the subgroup comparisons, particularly for different hospital levels and professional titles.

## Discussion

### *Surgeons' Awareness and Perspective on* *ERAS*

The current study found that more than half of orthopaedic surgeons had planned or implemented ERAS programs in their own hospitals. Doctors who highlighted the important influence of ERAS and thromboprophylaxis strategies more work at tertiary hospitals and/ or have had the titles of chief physicians. These findings might be associated with more ERAS exposure and medical education in high‐level hospitals. It is therefore necessary to provide education to younger surgeons in low‐level hospitals and smaller cities. Medical facilities and drug accessibility are more advanced in tertiary hospitals, which might facilitate ERAS implementation.

### *Choices of Pharmaceutical Therapy in Practice and Concerns*

Most surgeons agreed to consider the management of VTE prophylaxis after arthroplasty. However, conventional anticoagulants continue to have some obvious problem, such as delayed efficacy, regular monitoring, dose adjustment requirements, and high bleeding risk. LMWH has some advantages such as stable anticoagulant effect, quick onset, simple administration, low bleeding risk, and high safety^10^. It appears that LMWH is a reliable anticoagulant for patients undergoing replacement procedures; however, it can only be administered intravenously and subcutaneously, resulting in poor patient adherence after discharge^11^. Previous interventional studies revealed that NOACs are associated with a remarkable decrease in the incidence of VTE events when compared with LMWH, and have a similarly low risk of bleeding similar to LMWH^12^, ^13^, ^14^. A previous study reported that LMWH prophylaxis was commonly adopted during hospitalization after arthroplasty and that NOACs were more widely used after discharge because of their convenience^15^, and these results are consistent with our survey findings.

There is a high incidence of VTE within the 24 hours following major orthopaedic surgery; thus, doctors promote a primary focus on VTE prevention during this period. It is worth mentioning that anticoagulants can prevent VTE while introducing varying risks of bleeding complications^16^. The risk is directly related to their efficacy and administration time (i.e., greater effectiveness and earlier administration are associated with higher risk). Therefore, the risk and benefit should be carefully balanced when selecting the starting time of drug administration. According to the American College of Chest Physicians (ACCP) guidelines, LMWH administration is safe and reliable 12–24 h after surgery. We found that 37.7% of surgeons in this survey chose to start administration 12–24 h after surgery. On the other hand, the ACCP guidelines stated that the duration of treatment for VTE prevention should be at least 10–14 days, and it should be extended to 35 days after THA^17^. In this survey, the responses of about 56% of surgeons agreed with the duration recommended in the guidelines for VTE prevention in TKA patients. Additionally, 54.5% of surgeons considered using anticoagulants in THA patients for less than 4–5 weeks. Strong concern the survey respondents felt was about serious bleeding events after discharge, which is not adequately monitored during the time of patient follow‐up.

### *Potential Influence of Concomitant Use with Medications*

Perioperative blood loss and blood transfusion requirements associated with total joint arthroplasty remain primary concerns in orthopaedics^18^, ^19^, ^20^. Some studies have reported that intravenous TXA remarkably reduced blood loss after TKA/THA^21^, ^22^. However, the conclusions regarding its benefit or risk in clinical settings are inconsistent in different studies^23^, ^24^. According to the results of this survey, most surgeons believed that TXA has no effect on the initial administration time of NOACs. In addition, postoperative pain in patients with TKA/THA severely affects functional exercise ability. Analgesic management is greatly important for the recovery of joint function^25^, and It included the use of non‐steroidal anti‐inflammatory drugs (NSAIDs). However, NSAIDs might increase the risk of bleeding. Over 80% of survey respondents have concerned about the concomitant use of anticoagulants and NSAIDs. 

### *Limitations*

The present study has limitations. First, data were collected online, and data from undeveloped areas were harder to obtain. However, the low number of orthopaedic surgeons might have influenced the results. Second, the sample distribution was uneven, especially for the city‐level stratification, and this might have caused inconsistent results with regard to the other two stratifying variables.

### *Conclusion*

This study has investigated the concept of ERAS and thromboprophylaxis strategy and concludes that it is considered important among Chinese orthopaedic surgeons. The Bleeding risk with chemical thromboprophylaxis was still the greatest concern when choosing different antithrombotic regimens include its concomitant use of NSAIDs. Considerations for the implementation of ERAS program and antithrombotic regimens were closely related to different hospitals and professional titles. Therefore, CME is essential to improve the gap.

## Authorship Declaration

All authors listed meet the authorship criteria according to the latest guidelines of the International Committee of Medical Journal Editors, and all authors are in agreement with the manuscript.
